# Dihydrochalcones: Methods of Acquisition and Pharmacological Properties—A First Systematic Review

**DOI:** 10.3390/molecules24244468

**Published:** 2019-12-05

**Authors:** Monika Stompor, Daniel Broda, Agata Bajek-Bil

**Affiliations:** 1 Institute of Medical Sciences, University of Rzeszów, 35-959 Rzeszów, Poland; 2 Department of Biotechnology, Institute of Biology and Biotechnology, University of Rzeszów, 35-959 Rzeszów, Poland; danielbroda@wp.pl; 3 Faculty of Chemistry, Rzeszow University of Technology, 35-959 Rzeszów, Poland; abajek@prz.edu.pl

**Keywords:** antioxidants, dihydrochalcones, microbial transformations, phloretin, sweeteners

## Abstract

Dihydrochalcones are a class of secondary metabolites, for which demand in biological and pharmacological applications is still growing. They posses several health-endorsing properties and, therefore, are promising candidates for further research and development. However, low content of dihydrochalcones in plants along with their low solubility and bioavailability restrict the development of these compounds as clinical therapeutics. Therefore, chemomicrobial and enzymatic modifications are required to expand their application. This review aims at analyzing and summarizing the methods of obtaining dihydrochalcones and of presenting their pharmacological actions that have been described in the literature to support potential future development of this group of compounds as novel therapeutic drugs. We have also performed an evaluation of the available literature on beneficial effects of dihydrochalcones with potent antioxidant activity and multifactorial pharmacological effects, including antidiabetic, antitumor, lipometabolism regulating, antioxidant, anti-inflammatory, antibacterial, antiviral, and immunomodulatory ones. In addition, we provide useful information on their properties, sources, and usefulness in medicinal chemistry.

## 1. Introduction

Improvement of knowledge about biologically active nonnutritive food components and their positive effect on human organism is one of the main reasons for the development of functional food. In addition, more and more attention is paid to designing structurally defined analogues of bioactive molecules, which is particularly important for their medical use. A wide range of applications of natural compounds and their derivatives have become possible due to the variety of methods of their acquisition, including isolation from natural sources, mainly from plants, transformations involving natural catalysts (microorganisms, plants, and enzymes), as well as classical chemical synthesis.

The possibility of using flavonoids as food additives as well as active ingredients of cosmetics and medicines is limited due to their poor solubility in nonpolar solvents [1,2] and, thus, to their weak ability to penetrate cell membranes. Therefore, attempts are being made to functionalize these compounds [3,4,5,6].

In turn, the studies on the relationship between the structure and flavour of chalcones have shown that citrus flavonoids after their catalytic hydrogenation become sweet. This information gave rise to intensive research, which resulted in the development of industrial methods of synthesis of dihydrochalcones and their structural analogues [7,8]. The health benefits of dihydrochalcones include also anticancer [9,10], antioxidant, antibacterial [11], antidiabetic [12,13], estrogenic [14], anti-inflammatory [15], antithrombotic [16], antiviral [17], anti-herpes simplex virus [18], neuroprotective [19,20], and immunomodulation [21] properties. There are also some reports on the possibility of using glycosidic dihydrochalcone derivatives in dermoid-cosmetic preparations [22].

These compounds and other constituents of plants have been described many times. Much attention has been paid to natural flavones, flavanones, and chalcones, but in the literature, we have not found any comprehensive review of pro-health effects of dihydrochalcones and biotechnological methods of their synthesis, including biotransformations.

## 2. Dihydrochalcones from Natural Sources

The main natural source of dihydrochalcones are plants, in which they are biosynthesized by the phenylpropanoid pathway, which is a well-understood metabolic route [23]. In traditional medicine, they have been used for the treatment of liver cirrhosis, hypertension, diabetes, malaria, yellow fever, breast cancer, gastroenteric and inflammatory disorders, and dengue fever [24]. Initially, it was thought that the occurrence of dihydrochalcones was limited to only about 30 plant families. With the development of more sensitive analytical methods, it turned out that they are more widespread in nature [25]. The most common methods of dihydrochalcones isolation involve their extraction form leaves using such solvents as ethanol, diethyl ether, methanol, and hexane with dichloromethane or high-pressure extraction with supercritical CO_2_.

**Figure 1:**
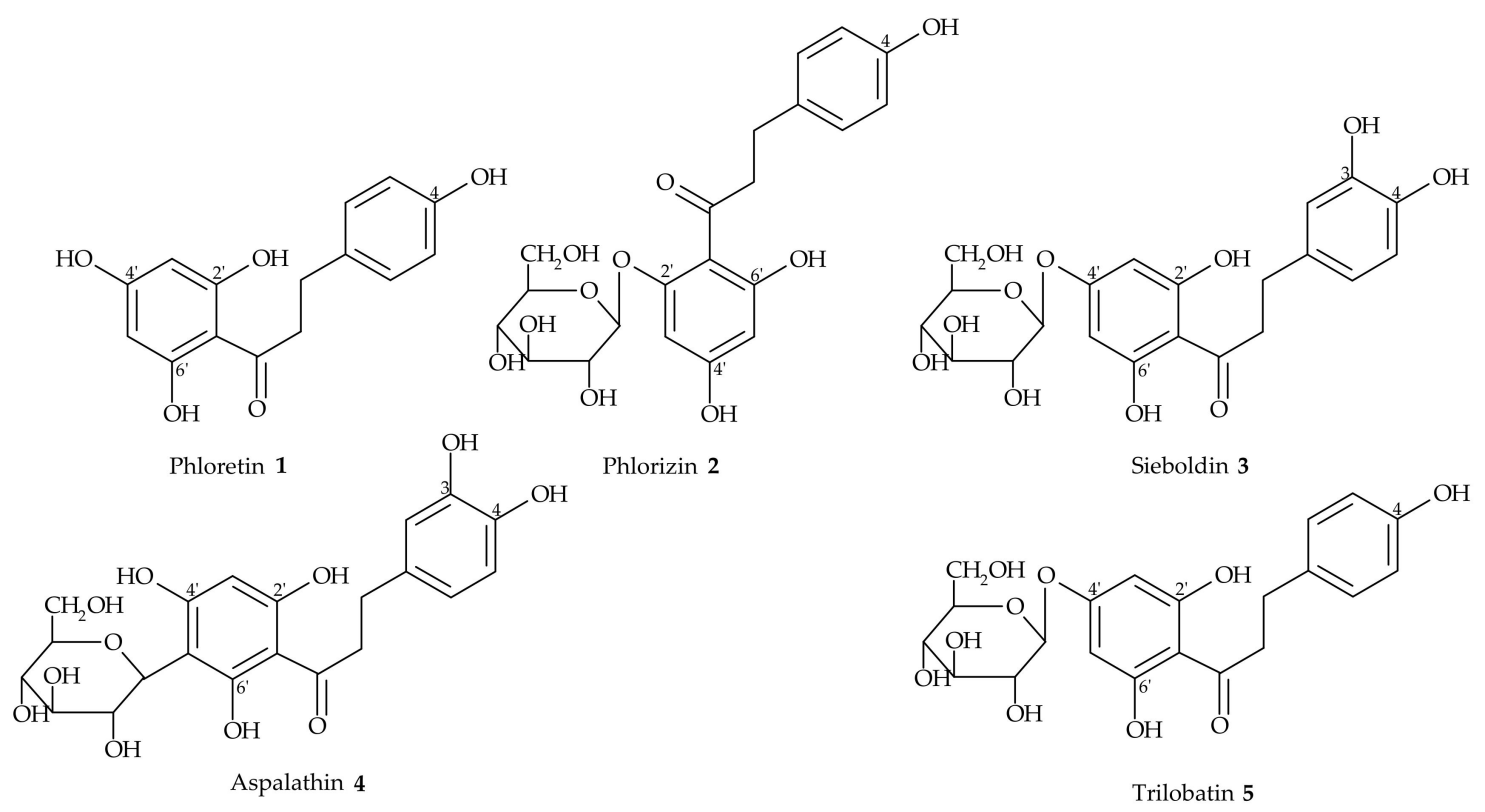
Dihydrochalcones from natural sources.

The richest source of natural dihydrochalcones in diet are apples. We can find these compounds also in apple leaves [26] and in hairy roots obtained through transgenesis [27]. Apple trees accumulate dihydrochalcones in very high concentrations—up to 14% of dry weight in leaves [28,29]. Their content in individual apple varieties is genetically determined [30]. Phloretin (**1**) is one of the best known apple dietary dihydrochalcones, characterized by the presence of 2,6-dihydroxyacetophenone pharmacophore. The composition of dihydrochalcones in various species and parts of apple trees is different. Flavonoid profile of apples also depends on the impact of pomace pretreatment and the extraction methods [31]. Some species of apples contain sieboldin (3-hydroxyphloretin 4′-*O*-glucoside; **3**) or trilobatin (phloretin 4′-*O*-glucoside; **5**) in place of phlorizin, or combinations of phlorizin (**2**), sieboldin (**3**), and trilobatin (**5**) (Figure 1).

As reported by Dixit et al. [32], the content of phlorizin (**2**) in the bark and leaves of apple species *Malus sikkimensis* may reach up to 12–13 mg/100 mg, which is about 90-fold higher than in fruits. Interestingly, phloretin was present only in the leaves (0.57 mg/100 mg). Furthermore, recently, dihydrochalcones were found also in cranberries [33], honeybush herbal tea (*Cyclopia* ssp.) [34], sofrito, and tomatoes [35]. They were also identified in such plants as *Esenbeckia grandiflora subsp. *brevipetiolata**, (roots) [36], Metrodorea nigra (fresh fruits) [37], *Leea indica* (Vitaceae) (leaves) [38], *Cytathostemma argenteum* (leaves and twings) [39], *Ulmus davidiana var. japonica* (Ulmaceae) (root bark) [40], *Glycyrrhiza iconica* (roots) [41], *Artocarpus altillis* [42], *Balanophora involucrata* [43], *Radula variabilis* [44], *Uvaria angolensis* (roots) [45], *Lithocarpus pachyphyllus* (Fagaceae) [46], *Lithocarpus polystachyus* (called Sweet Tea) [47], *Piper aduncum* [48], *Desmodium caudatum* [49], *Sinningia hatschbachii* [50], and *Colvillea racemosa* [51].

## 3. Chemical Synthesis of Dihydrochalcones

By chemical means, dihydrochalcones are obtained by regioselective reduction of carbon–carbon double bond in α,β-unsaturated ketones. One of the methods uses gaseous hydrogen, of which addition to the double bond is catalysed by ruthenium salts in dioxane at 80 °C [52].

Other methods use such common catalysts such as palladium [53], nickel, or iridium. Reference [54] developed a method of chemoselective reduction of α,β-unsaturated olefinic bond in chalcones using rhodium as a catalyst. Bagal et al. [55] applied complexes of palladium with N-heterocyclic carbenes (Pd-NHC) for chemoselective reduction of α,β-unsaturated carbonyl compounds, including chalcones. The use of carbene complexes of palladium is in accordance with the recommendations of “green chemistry” due to the possibility of their regeneration and reuse.

2’-Hydroxydihydrochalcones can also be obtained by chemical cleavage of the C ring in flavone, which occurs during catalytic hydrogenation [3]. Chen et al. [56] reported a high yield and selectivity of atmospheric hydrogenation of chalcone catalysed by recyclable thermoregulated phase-transfer Pd nanocatalyst. This method was characterised by the excellent selectivity (>99%) and high conversion of the substrates (99%).

Furthermore, such dihydrochalcones like brosimacutins H and I can be prepared by the enantioselective synthesis of cheap starting materials: hydroxyl-acetophenone and hydroxyl benzene formaldehyde [57].

The chemical synthesis of C-4-glucosylated isoliquiritigenin and its analogues and then the chemoselective reduction of the double bond in the obtained chalcones under hydrogenation conditions, using diphenyl sulfide (Ph_2_S) as an additive, were described recently [58].

The total synthesis of several naturally occurring dihydrochalcones (taccabulins B–E and evelynin) and 5-deoxyflavones, using Algar–Flynn–Oyamada oxidation and benzoquinone C–H activation, has been described by Sum et al. [59]. Dihydrochalcones can be also obtained from commercially available flavones, like quercetin or naringenin, in a five-step process with moderate yield (from 23% to 37%) [60].

In another method [61], *Bacillus stearothermophilus* maltogenic amylase (BSMA) was used for transglycosylation of neohesperidin dihydrochalcone. The obtained maltosyl-neohesperidin dihydrochalcone was 700 times more soluble in water but was, however, less sweet than the substrate, whereas Eichenberger et al. [62] proposed to employ *S. cerevisiae* as a microbial cell factory for de novo production of various dihydrochalcones of commercial interest. In turn, Gutmann et al. [63] described an efficient synthesis of glycosylated dihydrochalcones like phlorizin (**2**), davidigenin, and confusoside, using glycosyltransferase-catalysed cascade reactions.

The dihydrochalcone scaffold has recently been synthesized in a one-pot synthesis using Et_3_SiH in the presence of InCl_3_ via a sequential ionic hydrogenation reaction by switching the solvent [64].

## 4. Metabolism

**Scheme 1:**
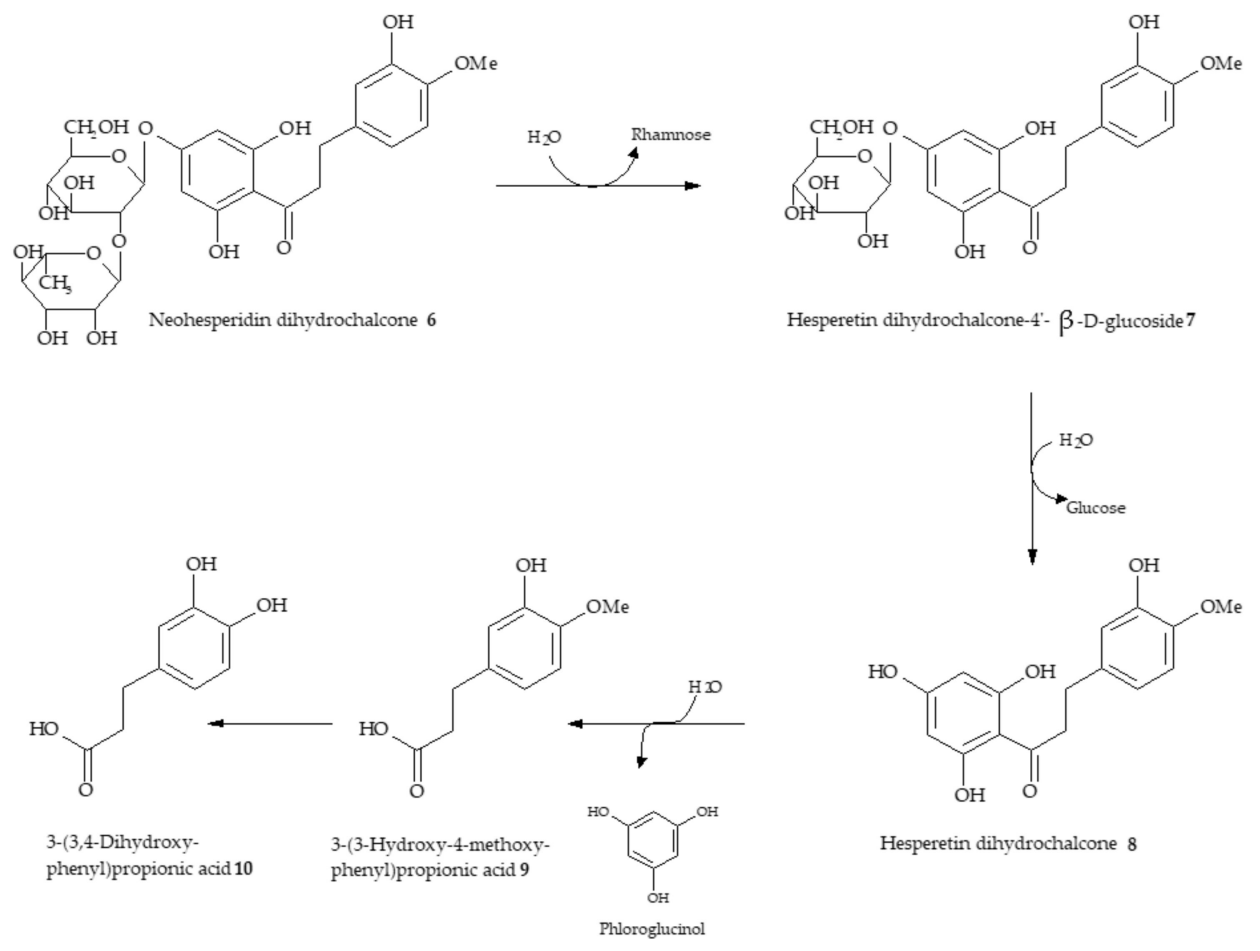
Degradation pathway of neohesperidin dihydrochalcone by human intestinal microbiota [65].

The degradative pathways of dihydrochalcones in vitro and in vivo were described in literature. It is known that the first step of transformation of neohesperidin dihydrochalcone (**6**) by the human intestinal microbiota is its deglucosylation to hesperetin dihydrochalcone 4′-β-D-glucoside (**7**) and subsequently to the aglycon hesperetin dihydrochalcone (**8**) (Scheme 1). Next, the latter is hydrolyzed to the corresponding 3-(3-hydroxy-4-methoxyphenyl)propionic acid (**9**) and probably to phloroglucinol [65].

**Scheme 2:**
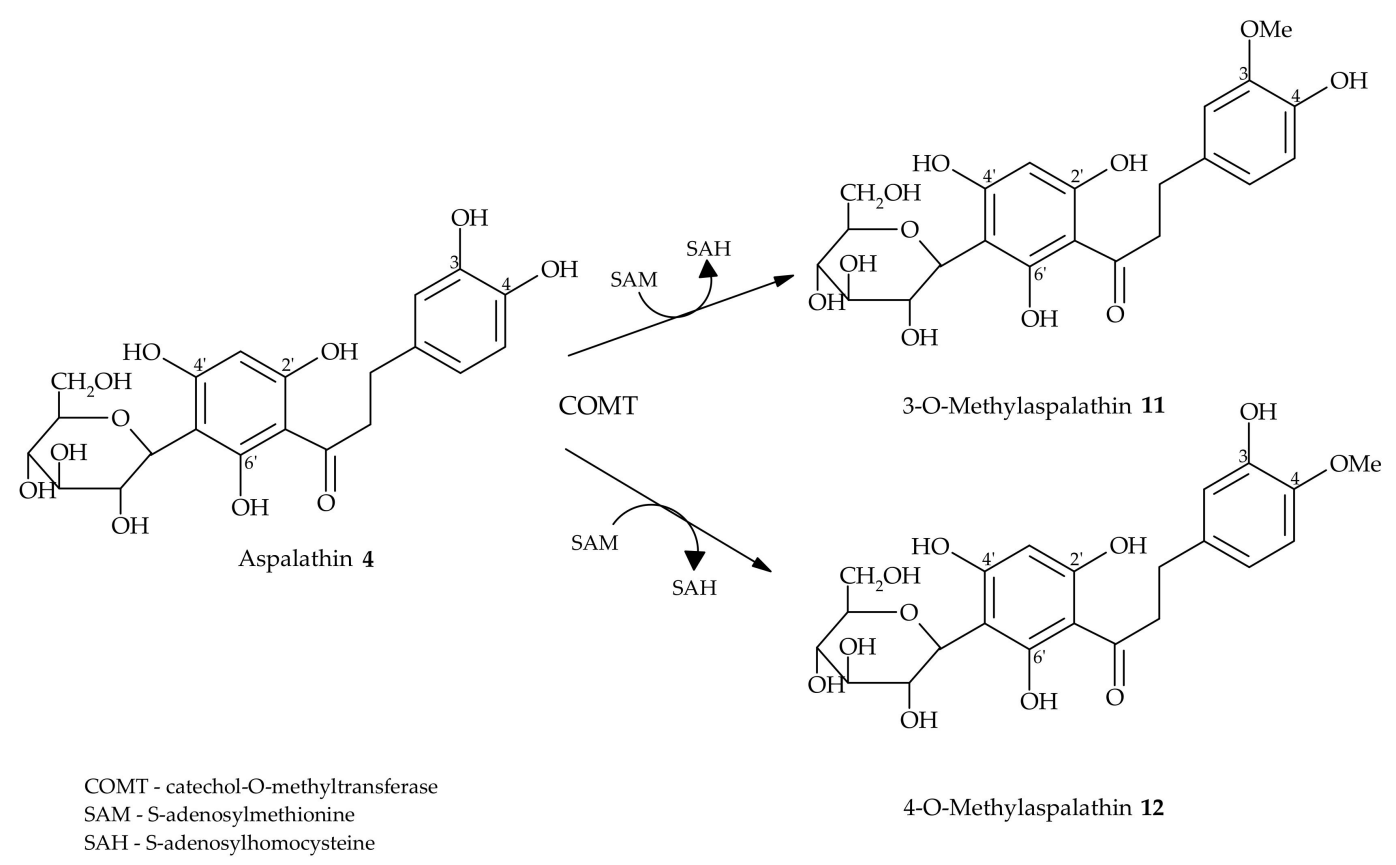
COMT-catalysed formation of two isomeric forms of *O*-methylated aspalathin metabolites.

In turn, Monge et al. [66] discussed the metabolism of phloretin (**1**) in rats and showed that both phloretin and phloridzin (**2**) were metabolised to phloretic acid and phloroglucinol. Courts and Williamson [67] noted that deglycosylation is not a prerequisite for C-glycosyl flavonoid absorption. The authors showed that flavonoid C-glycosides, like aspalathin (**4**), are methylated and glucuronidated in vivo in an intact form in humans (Scheme 2).

Kreuz et al. [68] performed the research on pigs, which were fed with rooibos tea extract for 11 days at an extremely high dose. Similarly, the results showed some intact metabolites in collected pig urine.

## 5. Microbiological and Enzymatic Hydrogenation of Chalcones

The chemical synthesis of dihydrochalcones from their corresponding flavones, flavanone glycosides, or chalcones is not economical because it requires a two-step synthesis, isolation of the corresponding product intermediate, and complicated hydrogenation procedure, utilizing high pressure.

An alternative to the chemical method of obtaining dihydrochalones is the use of whole cells of microorganisms. The majority of living organisms is able to hydrogenate the double bond. Biotransformation of chalcones results in similar yields as the chemical synthesis, under relatively mild process conditions. Whole cells of microorganisms can also catalyse the reduction of simpler aromatic ketones, aldehydes, imines, and unsaturated structures containing C=C double bond in the vicinity of strongly polar groups such as nitro, carbonyl, or hydroxyl ones. Additionally, these microbially synthesized products can be used as natural flavouring substances in accordance with the European Flavouring Regulation.

Due to the high selectivity of natural biocatalysts, production of dihydrochalcones by microbiological processes is a useful tool for the synthesis of chiral synthons that are used in the pharmaceutical industry. The obtained dihydrochalcones can be also used in food industry for production of multicomponent low-calorie sweeteners of natural origin as well as in pharmaceutical industry for production of natural flavonoid compounds showing anticancer, anti-inflammatory, antimalarial, and antioxidant properties.

**Scheme 3:**
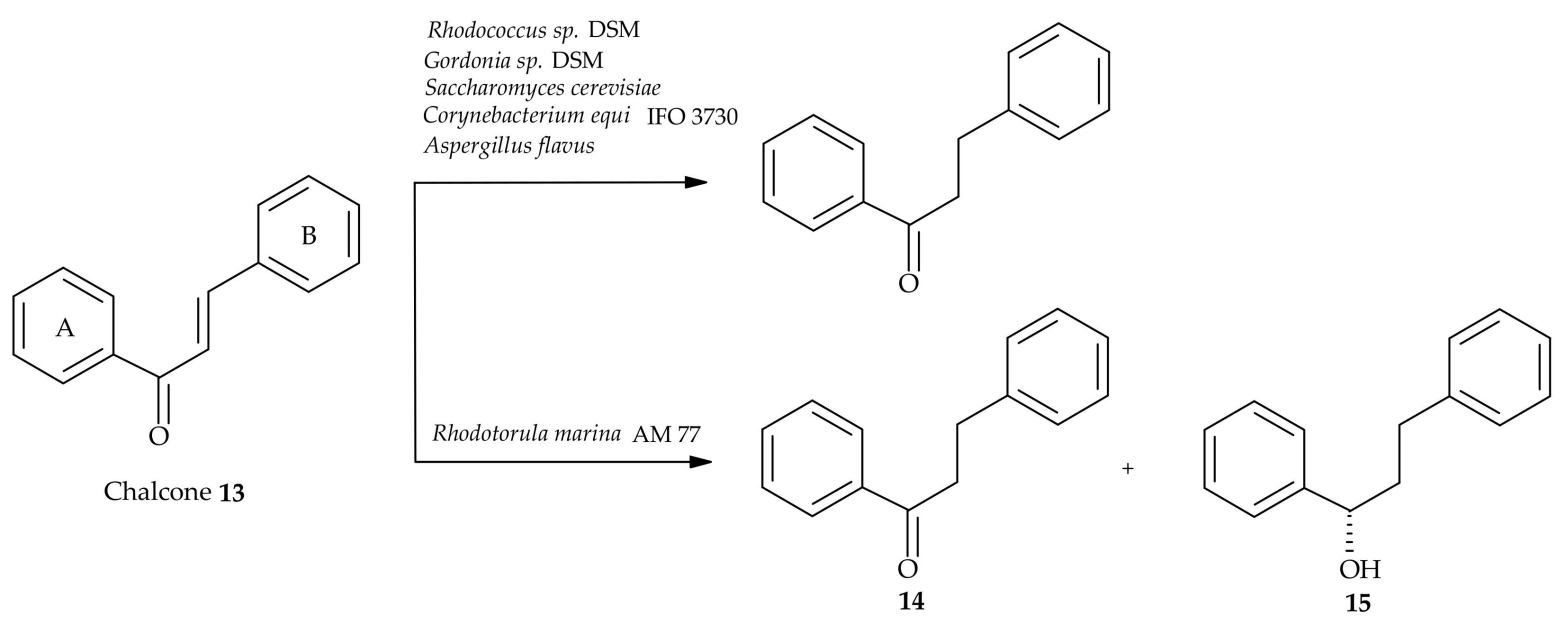
Bioreduction of chalcone (**13**) using variousmicroorganisms [69,70].

The first publication concerning microbiological hydrogenation of chalcones dates back to 1985. For transformation of 1,3-diaryl-2-propen-1-one (chalcone **13**) derivatives, whole cells of the genus *Corynebacterium equi* IFO 3730 were used (Scheme 3) [69].

**Scheme 4:**
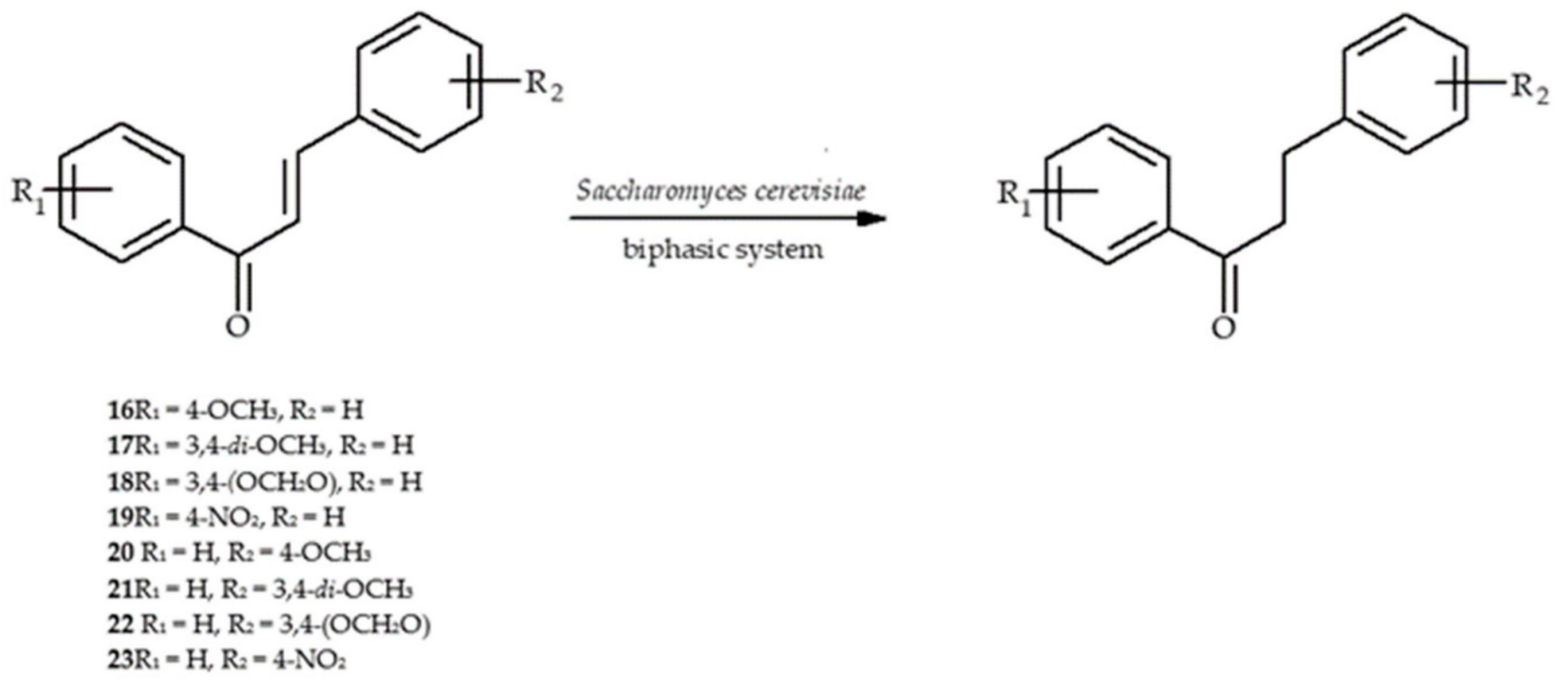
Biohydrogenation of chalcones (**16**–**23**) catalyzed by *S. cerevisiae* [70].

In another study, chalcone derivatives containing either nitro or alkoxy groups in aromatic rings (**16**–**23**) were studied. The products of highly efficient chemoselective microbiological hydrogenation in the presence of *Saccharomyces cerevisiae* industrial yeast strains were obtained. Biotransformations were carried out in a water-organic solvent two-phase system (Scheme 4) [70].

**Scheme 5:**
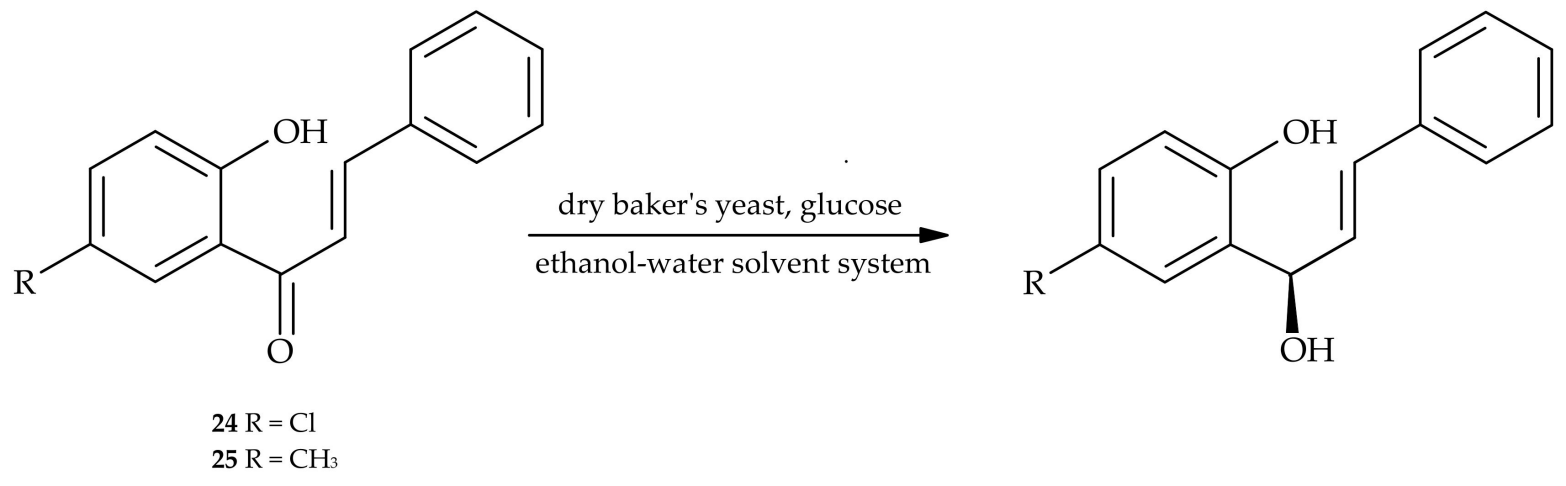
Bioreduction of chalcones in an ethanol–water system [71].

The team of Pathan et al. [71] used baker’s yeast *Saccharomyces cerevisiae* for stereoselective reduction of chalcones (**24** and **25**), β-diketones, and their derivatives containing various substituents in ring A (Scheme 5). They optimized the reaction conditions, including substrate concentration, temperature, pH, biomass amount, reaction time, and solvents. It has been proven that whole microbial cells reduce substrates independently of regeneration of the carbonyl reductase cofactor (NADPH). The reaction was most effective in the two-phase systems: water-strongly polar organic solvent.

**Scheme 6:**
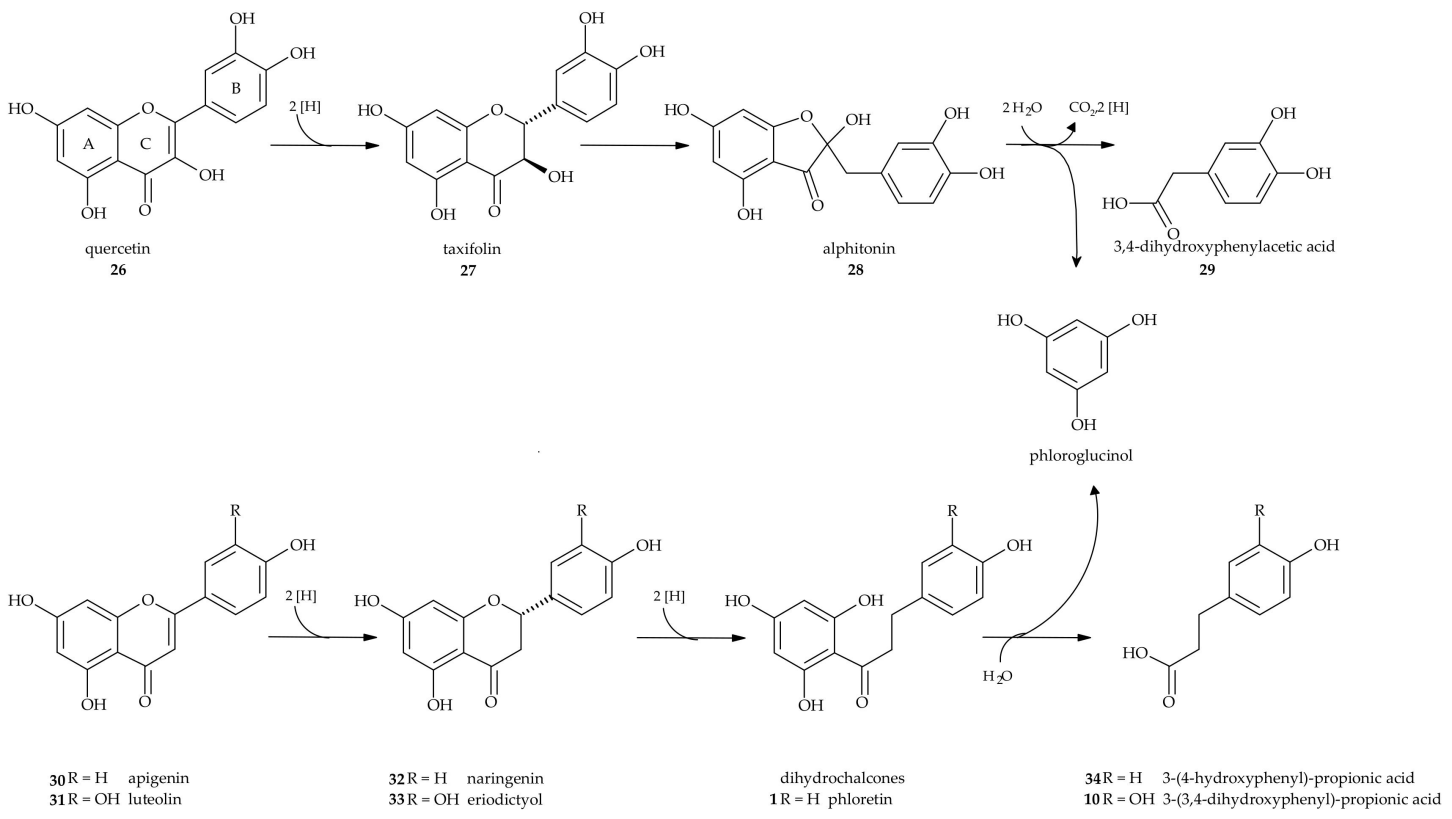
Pathways of quercetin (**26**), apigenin (**30**), and luteolin (**31**) degradation by *C. orbiscindens* [73].

In similar experiments carried out with chalcone derivatives, live culture of the fungus *Aspergillus flavus* [72] was used. Products of microbiological hydrogenation of olefinic bond in chalcones were also obtained by transformation of flavones: apigenin and luteolin, using *Clostridium orbiscindens strain* (Scheme 6) [73].

**Scheme 7:**
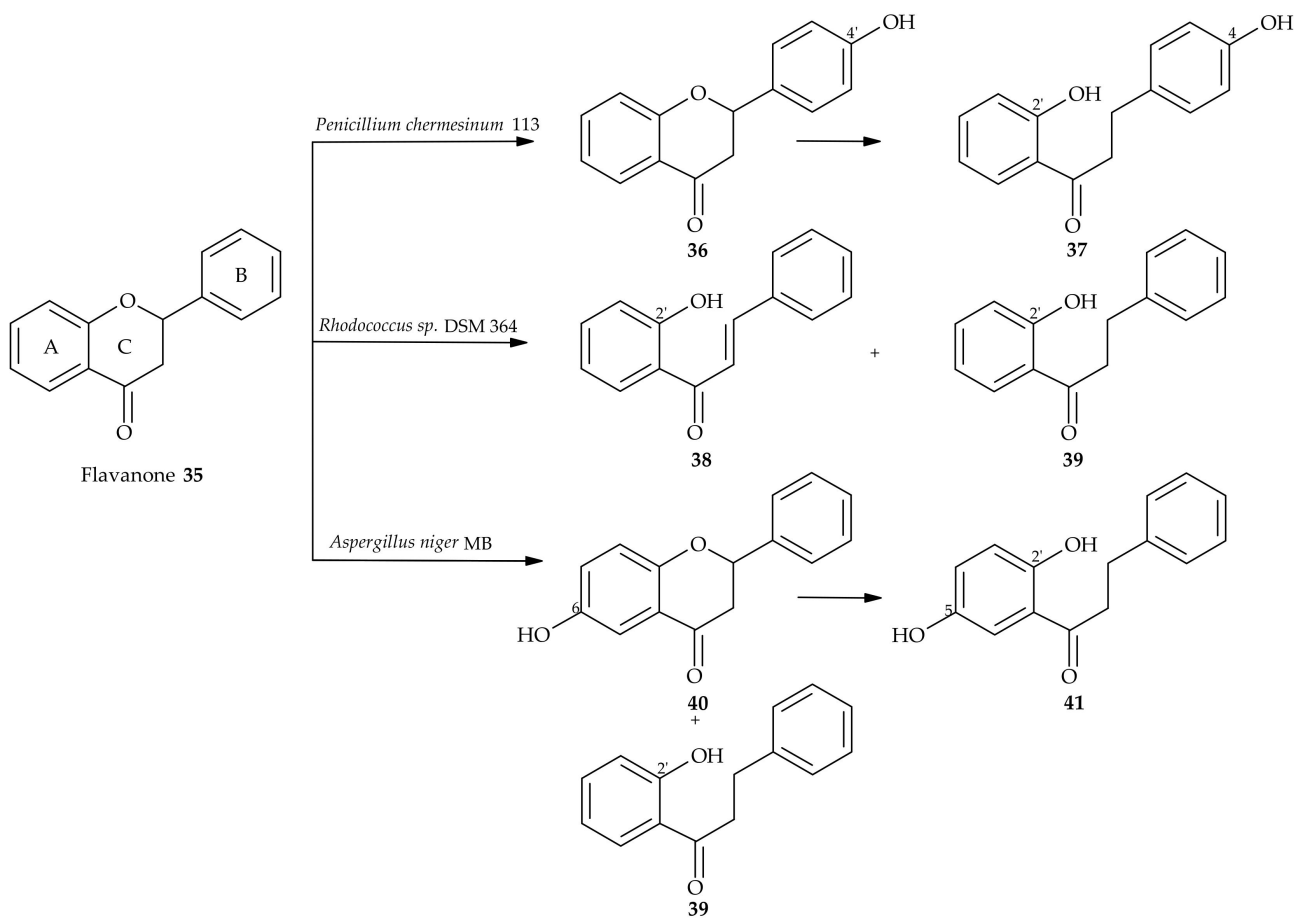
Biotransformation of flavanone (**35**) catalyzed by selected bacteria and fungi [74].

There is also a known method of obtaining 2′-hydroxydihydrochalcones by the microbiological cleavage of the C ring in flavanone (**35**) using filamentous fungi of the genus *Aspergillus* (Scheme 7). Apart from the product of the C ring cleavage (2′-hydroxydihydrochalcone (**39**), 7% yield), the product of hydroxylation in the A ring was also obtained (6-hydroxyflavanone (**40**), 18% yield), which in the further stage underwent transformation to 2′,5′-dihydroxydihydrochalcone (**41**) (6% yield) [74]. Similarly, as a result of biotransformation of flavanone (**35**) by *Penicillium chermesinum* 113 strain, a hydroxy derivative of the substrate was obtained in the first stage (4′-hydroxyflavanone (**35**), 11.8% yield), and then 2′,4-dihydroxydihydrochalcone (**37**) (36.5% yield) was obtained as a result of the C ring cleavage [74]. Also, the strain *Rhodococcus sp*. DSM 364 was capable of cleaving the C-ring of flavanone (**35**), which led to 2′-hydroxychalcone (**38**) and, next, to 2′-hydroxydihydrochalcone (**39**). The hydroxyl derivative of flavanone—naringenin (5,7-dihydroxyflavanone (**32**))—was resistant to biotransformation by this strain.

**Scheme 8:**
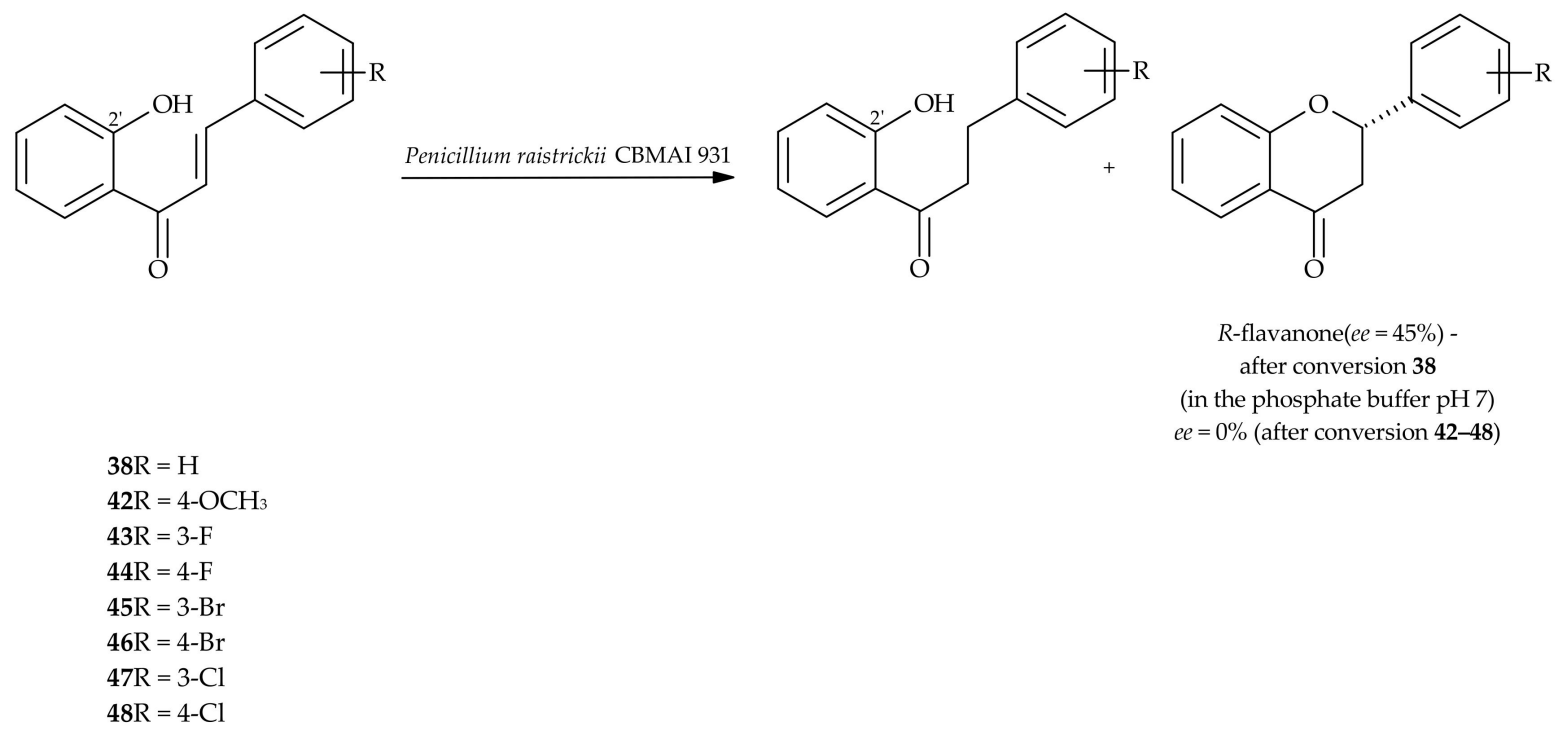
Chemoselective hydrogenation of 2′-hydroxychalcones (**38**, **42**–**48**) by *P. raistrickii* [75].

In turn, a biotransformation of 2′-hydroxychalcone (**38**) and its halogenated derivatives (**42**–**48**) in the marine-derived fungus *Penicillium raistrickii* CBMAI 931 led to the regio- and chemoselective hydrogenation (Scheme 8) to give the products with good conversion (78–99%) and isolated yields between 31 and 65%. The formation of flavanones was low (3–14%). 2′-Hydroxydihydrochalcone (**39**) with 72% conversion and *R*-flavanone with 18% conversion were obtained after 7 days of reaction in phosphate buffer (pH 7) [75].

**Scheme 9:**
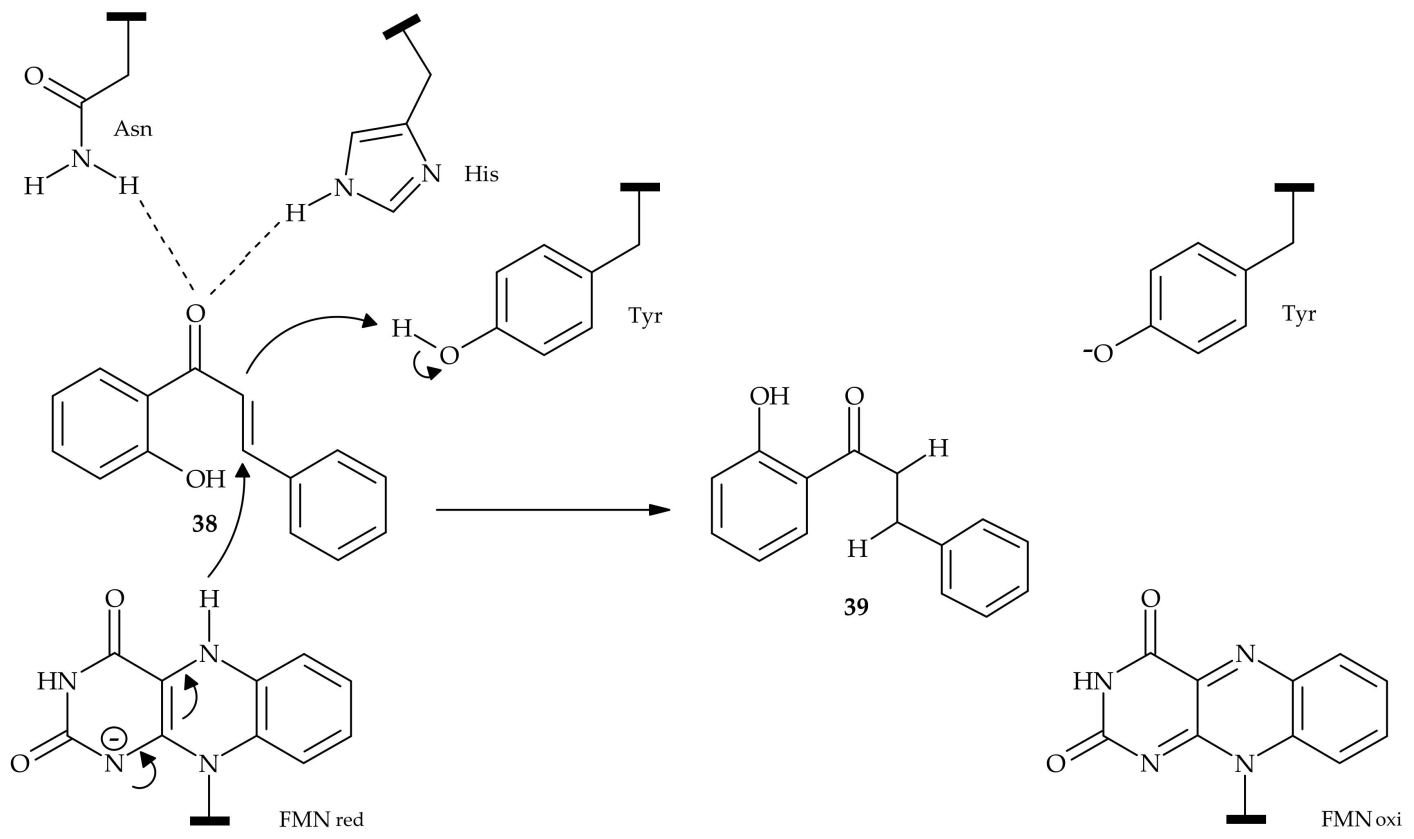
Reaction sequence proposed for the chemoselectve reduction of C=C of 2′-hydroxychalcone (**38**) mediated by ene-reductases [75].

The hydroxychalcones containing a fluorine atom were hydrogenated with higher yields than the analogous 2′-hydroxychalcones with bromine or chlorine atoms in the same positions of the aromatic B ring. It may be related to the strong hydrogen bonds that fluorine-containing compounds can form with the amino acids, such as histidine and asparagine residues, which are present at the active sites of the ene-reductases that are responsible for catalyzing the reaction (Scheme 9).

**Scheme 10:**
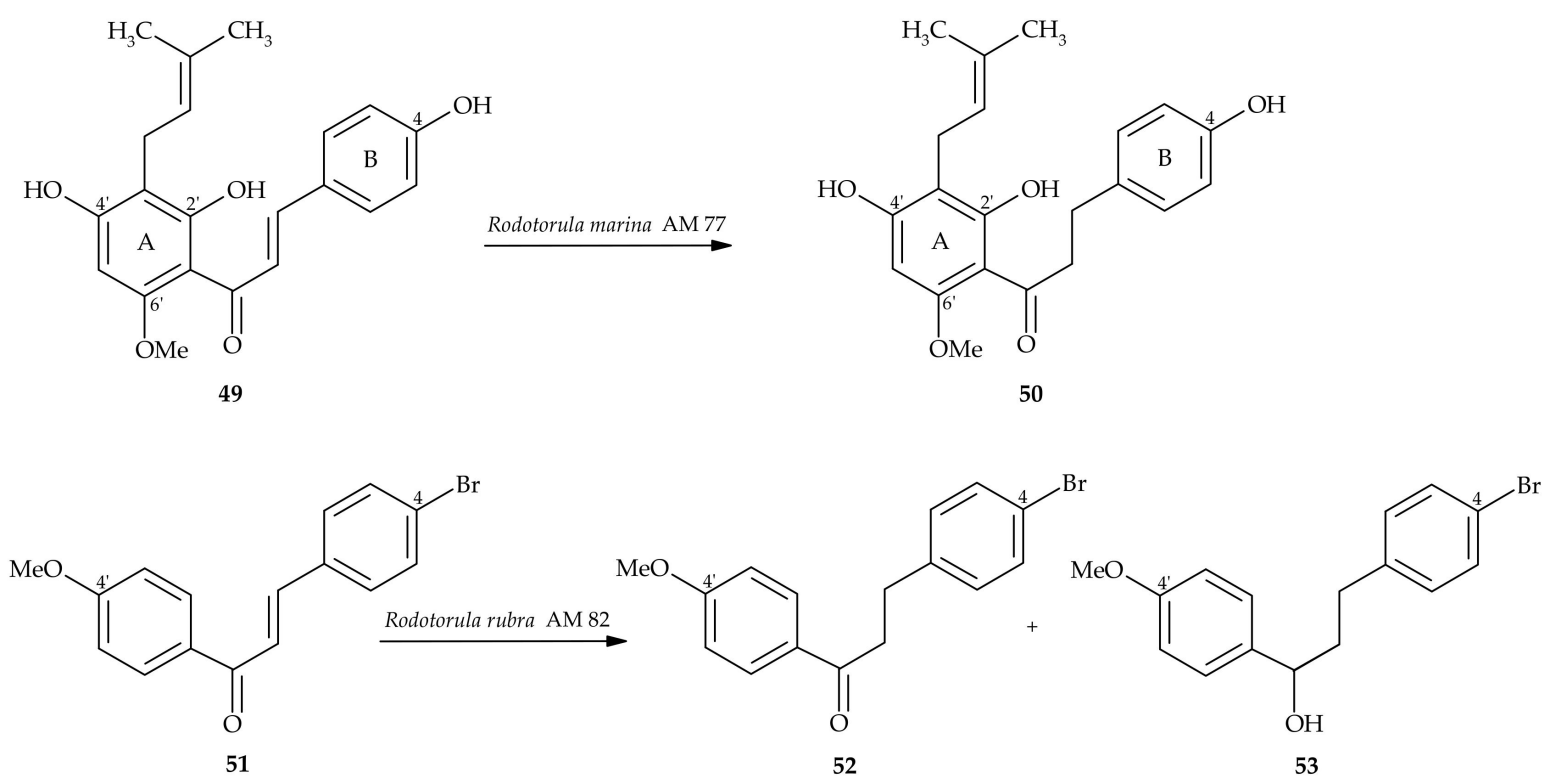
Biotransformation of chalcones (**49** and **51**) by *Rhodotorula* strains [76].

Yeast *Rhodotorula* possesses specific enzymes capable of hydrogenating chalcones in good yield (Scheme 10) [76]. The strain *Rhodotorula rubra* AM 82 biotransformed chalcones into dihydrochalcones and respective secondary alcohols. In addition, some other species of yeast like *Saccharomyces* and *Yarrowia* posses a high ability to reduce the double bond in chalcones and their derivatives, which was described recently [77]. The substrates in these reactions included predominantly hydroxy- and methoxyderivatives of chalcones and compounds containing furan and thiophene substituents.

**Scheme 11:**
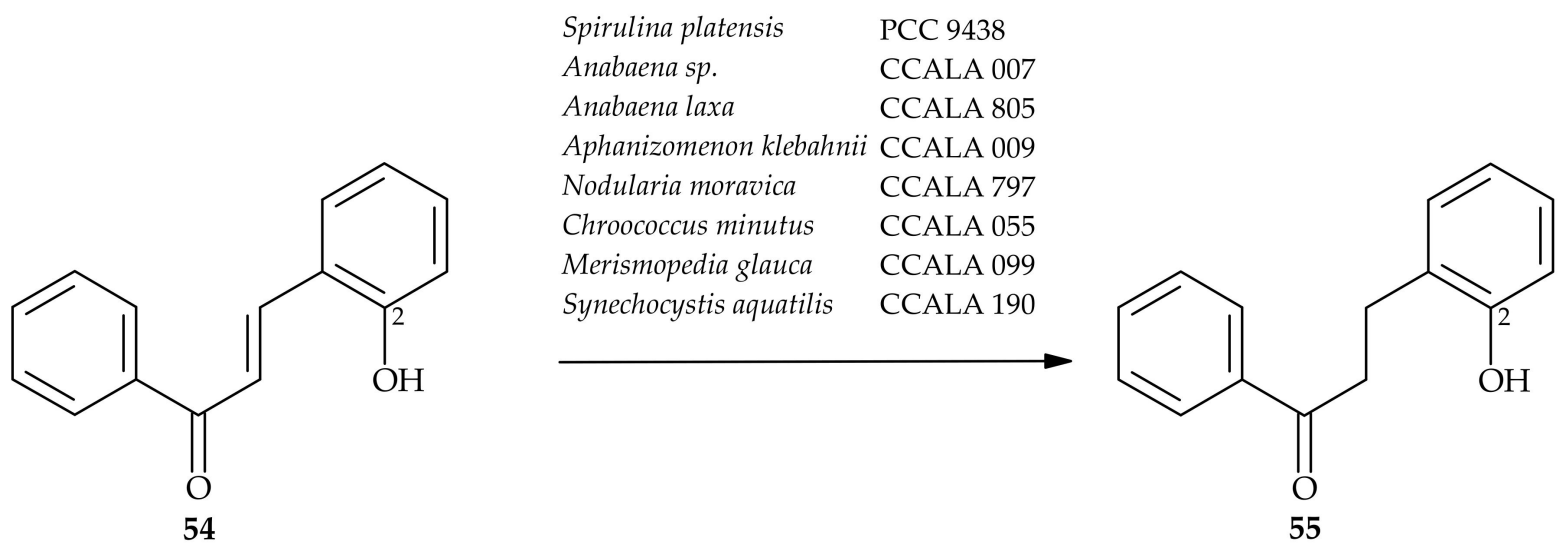
Biotransformation of 2-hydroxychalcone (**54**) by cyanobaceria [78].

Such an activity has also been reported in relation to cyanobacteria (Scheme 11) [78]. The bioconversion of 2-hydroxychalcone (**54**) proceeded with high efficiency. Most of the tested cyanobacterial strains converted this compound into its hydrogenated derivative (**55**) in nearly 100% yield.

**Scheme 12:**
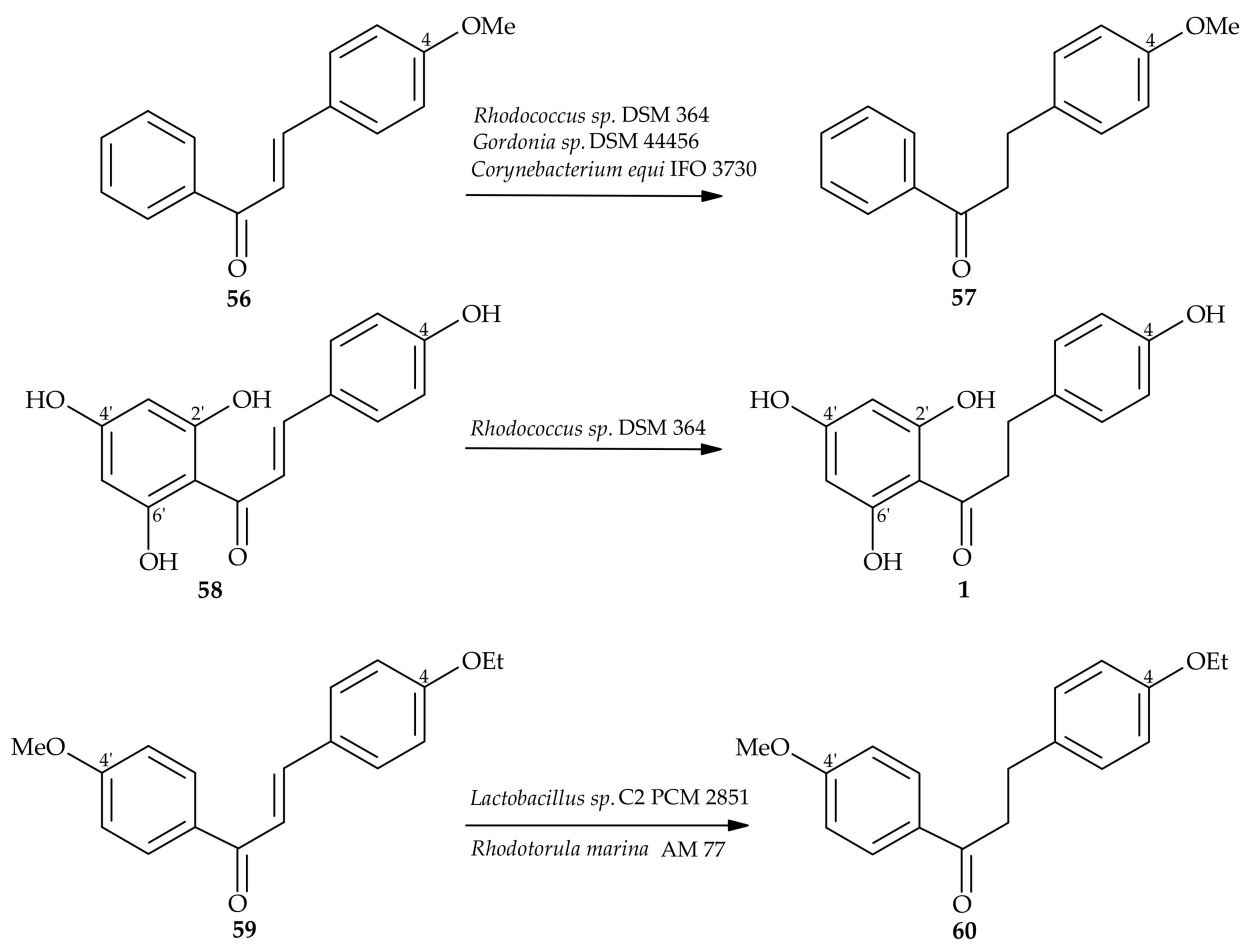
Biotransformation of chalcones using select biocatalysts [69,79,80].

Chalcones can be also converted to dihydrochalcones using bacterial strains of the genera *Rhodococcus*, *Gordonia*, and *Lactobacillus* (Scheme 12) [79,80].

In turn, Klingel et al. [81] modified and applied a recombinant dextransucrase from *Lactobacillus reuteri* TMW 1.106 to glucosylate various flavonoids and flavonoid glycosides. An efficient glucosylation was achieved for quercetin (**26**) and its glycosides—quercetin-3-*O*-*β*-glucoside and rutin—whereas the efficiency of glucosylation for neohesperidin dihydrochalcone was low. Additionally, it was noted that the glycosyl substituents such as β-glucose, rutinose, or neohesperidose were glucosylated at varying positions.

Lin et al. [82] developed a new methodology for production of natural sweetener trilobatin in a two-step enzymatic process, in which naringin was hydrogenated into naringin dihydrochalcone, which was followed by removal of the rhamnosyl group of naringin dihydrochalcone by enzymatic hydrolysis using immobilised α-l-rhamnosidase as the catalyst. The optimized conditions involved using a very low loading of the α-l-rhamnosidase catalyst at 60 °C in a neutral aqueous buffer solution within 2 h. The overall yield of 96% was achieved.

## 6. Pharmacological Properties of Dihydrochalcones

Dihydrochalcones have beneficial therapeutic effects on the human body, proven in vitro and in vivo (Table 1). Studies on their pharmacological potential include antidiabetic, immonomodulatory, antiviral, cardio, and hepatoprotective activity. Some studies already spotted the significance of chalcones and dihydrochalcones as antimicrobial agents. However, the literature data concerning the antimicrobial activity of this group of compounds are still scarce.

Many data show that flavonoids, including dihydrochalcones, are excellent antioxidants that can scavenge free radicals through a variety of mechanisms.

On the other hand, anticancer and antimetastatic effects have also been ascribed to these metabolites. They may influence the development of cancer, as a result of several important mechanisms: regulation of angiogenesis, modification of the antitumor response, and direct influence on tumour cell proliferation and apoptosis. Additionally, a correlation was found between total polyphenolic content of crabapple fruit extracts and their antioxidant and antiproliferative effects.

However, the biological activity of dihydrochalcones depends mainly on their chemical structure and the presence of sugar residues attached to the molecule.

Some similarities in the structures of phloterin’s analogue phlorizin and 17-β-estradiol, both of which have two aromatic nuclei and hydroxyl substitution in the ring, may be the reason for the fact that phlorizin shows both estrogenic or antiestrogenic effects and can bind to estrogen receptors. This property may be used in anticancer therapy, for example, in the case of breast cancer, where cell proliferation may be induced by estrogen-like substances, and in other estrogen-related diseases [83]. There is also some evidence that the anticancer effects are enhanced by the antioxidant and antiinflammatory properties of dihydrochalcones.

Moreover, anti-hyperglycaemic and anticholinesterase effects of dihydrochalcones have been reported. According to the latest information, some of the natural dihydrochalcones may be potentially used as safe nutraceuticals to protect against metabolic disease-related complications. 

Dihydrochalcones may be also good candidates for drugs against Alzheimer’s disease, which was confirmed by the virtual screening method (computer simulation and experiment) [84].

The interest in natural sweeteners is constantly growing among nutrition and food production specialists as well as among doctors and nutritionists. This is due to the fact that consumption of synthetic sweeteners causes many civilization diseases, such as atherosclerosis, obesity, and diabetes.

Dihydrochalcones are a part of our daily diet. We can find them in plants, or we can synthesize them by chemical and enzymatic methods. Naringin dihydrochalcone and neohesperidin dihydrochalcone, which may be also chemically synthesized from citrus flavanones, are low-calory sweeteners with one and twenty times the sweetness of saccharin on a molar basis, respectively [85]. Neohesperidin dihydrochalcone was also approved as a food additive in Europe (E959 code) [86].

Bearing in mind the demand from the pharmaceutical and medical industry for preparations based on natural compounds that are effective in prevention and therapy of various diseases, further research on pharmacokinetic and pharmacodynamic aspects is encouraged to improve new production and delivery methods and to achieve feasible dihydrochalcone-based clinical formulations.

## 7. Dihydrochalcones as the Scaffolds for Drug Development

Dihydrochalcones are naturally occurring α,β-unsaturated carbonyl compounds, which are the key precursors for the biosynthesis of other flavonoids and for the synthesis of many biologically valuable heterocyclic compounds such as benzothiazepines; pyrazolines; derivatives containing other ring type of N-, O-, and S-heterocycles; and flavones [142]. The biological potential of chalcone derivatives may be increased by the presence of nitrogen, oxygen, or sulfur-containing heterocyclic scaffolds.

In turn, the biotransformation products, such as 2’-hydroxydihydrochalcone, may be used as substrates for synthesis of antiarrhythmic drugs used for restoring normal heart rhythm, e.g., propafenone, which additionally shows high anticancer activity [143].

Synthesis of a series of 2′-hydroxy chalcones and aurones containing isoxazole and pyrazole functions and biological evaluation of their antioxidant and antibacterial activity were performed by Irshad et al. [144].

A novel prenylated dihydrochalcone named adunchalcone was extracted from *Piper aduncum* L. (Piperaceae) by Dal Picolo et al. [145]. In further perspectives, this molecule may be used as a scaffold for the design of novel antileishmanial drugs for skin diseases.

Chakraborty et al. [146] described the multifunctional neuroprotective activity of neohesperidin dihydrochalcone as a potential novel scaffold for therapeutics against neurodegenerative disorders, such like Alzheimer’s disease. Jin et al. [147] noted that some natural dihydrochalcones might be good drug candidates to treat Alzheimer’s disease. It has been shown that a compound extracted from *Daemonorops draco* tree inhibited amyloid-β peptide aggregation and stopped production of the toxic fibrillary aggregates, which are responsible for the formation of neurofibrillary tangles, one of the main symptoms of dementia [146]. There are also some reports on synthesis of dihydrochalcone derivatives as potential sweeteners [148].

Some reports show that the glucuretic effect resulting from SGLT2 inhibition contributes to reduction of hyperglycaemia and also assists weight loss and blood pressure reduction. This phenomenon was later used for the development of synthetic antidiabetic drugs [149], three of which have been approved by the Food and Drug Administration (FDA) and European Medicine Agency (EMA) [150].

Inhibiting glucose reabsorption by sodium glucose co-transporter proteins (SGLTs) in kidneys is a relatively new strategy for treating type-2 diabetes. Selective inhibition of SGLT2 over SGLT1 is critical for minimizing adverse side effects associated with SGLT1 inhibition.

In other studies, Jesus et al. [150] synthesized a library of C-glucosyl dihydrochalcones and their dihydrochalcone and chalcone precursors and tested them as SGLT1/SGLT2 inhibitors using a cell-based fluorescence assay of glucose uptake. These results point toward the discovery of structures that will be potent and highly selective inhibitors of SGLT2. Due to the high potential of dihydrochalcones, research in this area should be continued. The SGLT2 inhibitors such as dapagliflozin, canagliflozin, and tofogliflozin are also available in many countries as pharmaceutical drugs to treat diabetes.

## 8. Conclusions

Recently, much attention has been paid to the search for new compounds that show properties conducive to maintaining health and to proper functioning of the body. Flavonoids are examples of substances that may be included in nutraceuticals. Microbial transformations of flavonoid compounds can be a natural alternative to chemical synthesis of dihydrochalcones. The biotransformation processes may be useable to modify the compound structures in order to improve their biological properties and to increase lipophilicity and bioavailability. Another natural method of obtaining of these compounds is their isolation from plants.

This review article contains sections with general information about the biological activity of dihydrochalcones as well as documented results confirming their potential usefulness in alternative medicine. This review can be a guide for researchers dealing with innovative treatment methods that use natural dietary components as well as for planning new methods of obtaining more effective molecules containing the dihydrochalcone skeleton.

## Figures and Tables

**Table 1:** Comparison of biological activity of dihydrochalcones in various experimental models.

Therapeutics	Compound	Treatment and Methods	Targets and Effects	Experimental Model	Ref.
Anticancer	evelynin A	SRB assay	antiproliferative activity IC_50_ = 6.3 ± 0.7µM (HeLa) IC_50_ = 4.7 ± 0.4 µM (PC-3)	In vitro, HeLa (cervical carcinoma), A549 (lung cancer), and PC-3 (prostate cancer)	[87]
	evelynin B		antiproliferative activity IC_50_ =8.8 ± 0.5 µM (HeLa) IC_50_ = 8.3 ± 0.6 µM (A549) IC_50_ = 5.0 ± 0.8 µM (PC-3) inhibits tubulin polymerization		
	aspalathin	0.2–1.0 µM	Co-treatment with aspalathin attenuates doxorubicin-induced cardiotoxicity by improving endogenous antioxidant levels and mitochondrial membrane potential while inhibiting reactive oxygen species production and cellular apoptosis.	In vitro, H9c2 cardiomyoblasts	[88]
	zornioside	0.39–50 µg/mL MTT assay	IC_50_ = 37.26 µM	In vitro, HL60 (leukemia cells)	[89]
	sakenins F and H		PC_50_ values of 8.0 µM and 11.1 µM	In vitro, PANC-1 human pancreatic cancer cells	[90]
	2′,6′-dihydroxy- -4′4-dimethoxydihydrochalcone, 2′,6′-dihydroxy- -4′-methoxydihydrochalcone, and 2′,4′,6′-trihydroxydihydrochalcone	MTT assay 50–100 μM	Augment TRAIL (tumor necrosis factor-related apoptosis-inducing ligand)-induced apoptosis and cytotoxicity	In vitro, LNCaP (human hormone-sensitive prostate cancer)	[91]
	2′-dihydroxy-4,4′-dimethoxydihydrochalcone	8–32 µM 12–48 mg/kg	Antiproliferative activity, suppresses cell proliferation, induces excess of reactive oxygen species (ROS) accumulation, and inhibits the invasion and migration ability of MKN45 cells. Upregulates the autophagy-related proteins Beclin-1, Atg5, and Atg7; induces formation of Autophagosomes and regulatory role of ROS/MEK/ERK signaling pathways. In vivo inhibits the growth of MKN45 xenograft tumors in nude mice and suppresses Ki67 expression.	In vitro, MKN45 (human gastric cancer) In vivo, BALB/C nude mice	[92]
	conjugates of phloretin (Pht) and phlorizin (Phl) with gold nanoparticles (AuNPs)		Fourty-five-fold increase in the efficacy in the antineoplastic activity of Pht-AuNPs over pure phloretin and 4.49-fold increase in efficacy of Phl-AuNP over pure phlorizin.	In vitro, HeLa (cervical carcinoma)	[93]
	phloretin	0–60 µM 10–20 mg/kg	Marked suppression of invasion and migration through downregulation of matrix metalloproteinase (MMP)-2, MMP-3, and cathepsin Sin human SiHa cervical cancer cells. Suppresses lung metastasis and tumor growth.	In vitro, SiHa (cervical cancer), HeLa, and CaSki (cervical cancer) In vivo, BALB/c AnN.CgFoxn^nu^/Crl Narl mice	[94]
		0.1–0.2 mM	Induces apoptosis by the inhibition of glucose transmembrane transport and internucleosomal DNA fragmentation	In vitro, B16 (mouse melanoma 4A5 cells)	[95]
		20–100 µM	Inhibits cancer cell proliferation and migration by increasing the ROS production in the cells	In vitro, PC-3 and DU-145 (human prostate cancer)	[96]
		60–100 µg/mL	Induces cancer cellapoptosis via a mitochondrial-dependent pathway. Downregulates the antiapoptosis-associated molecule B-cell lymphoma 2 (bcl-2) and increases the levels of the apoptosis-associated molecules bcl-2-like protein 4 and tumor protein p53. Affects the expression of apoptotic protease activating factor-1.	In vitro, EC-109 (human esophageal cancer)	[97]
		MTT assay, DAPI, annexin V/PI, Transwell assay, and Western blotting. 0–16 µM	Inhibits cell proliferation. IC_50_ = 8.0 µM (AGS) IC_50_ = 16.0 µM (MGC80-3, BGC-823, SGC-7901, SNU-5) IC_50_ = 32.0 µM (SNU-1, SNU-5, SNU-16, RF-1) IC_50_ > 60 µM (SNU-16, GES- 1)	In vitro, MGC80-3, BGC-823, SGC-7901, AGS, SNU-1, SNU-5, SNU-16, RF-1, and GES-1 (gastric cancers)	[98]
	3‴-methoxy-6″-*O*-feruloy-4′-*O*- -glucopyranosyl-phloretin	MTT assay	IC_50_ = 39.79 ± 5.72 µg/mL (A549) IC_50_ = 59.28 ± 5.06 µg/mL (BEL7402) IC_50_ = 49.36 ± 3.04 µg/mL (HepG2) IC_50_ = 65.09 ± 2.77 µg/mL (HT-29) Glycoside moiety bound to the phloretin structure decreases its anticancer activity (e.g., in trilobatin)	In vitro, A549 (lung cancer), BEL 7402 (liver cancer), HepG2 human ileocecal cancer cell line, and HT-29 (colon cancer)	[99]
	3-hydroxyphloretin		IC_50_ = 39.83 ± 4.23 µg/mL (A549) IC_50_ = 45.17 ± 8.02 µg/mL (BEL7402) IC_50_ = 37.79 ± 4.04 µg/mL (HepG2) IC_50_ = 35.37 ± 2.53 µg/mL (HT-29)		
	α,β-dihydroxanthohumol	SRB assay 0.1–100 µg/mL	IC_50_ = 9.15 ± 0.62 µM (MCF-7)) IC_50_ = 14.73 ± 3.88 µM (PC-3) IC_50_ = 74.41 ± 23.44 µM (HT-29)	In vitro, MCF-7 (breast cancer), PC-3 (prostate cancer), and HT-29 (colon cancer)	[100]
	4,2′,4′-trihydroxy-6′-methoxy-3′(2″-hydroxybenzyl)dihydrochalcone, 2′,4′-dihydroxy-4,6′-dimethoxy-3′(2″-hydroxybenzyl)dihydrochalcone		Antitumor activity IC_50_ = 2.02–20.03 µg/mL	In vitro, KB (epimermoid carcinoma), MCF-7 (breast cancer, and NCl-H187 (lung cancer)	[101]
Antidiabetic	panduratin isopanduratin	MG–BSA assay	Antiglycation agents, α-glucosidase inhibition	In vitro, bovine serum albumin and α-glucosidase (from rat intestine)	[102]
	3′-O-β-d-glucopyranosyl α,4,2′,4′,6′-pentahydroxy-dihydrochalcone	25, 50, and 100 mg/kg diabetic nephropathy	Antiglycation compound inhibits protein glycation and decreases accumulation of advanced glycation end products (AGEs).	In vivo, STZ (streptozotocin)-induced diabetic mice (C57BL/6)	[103]
	phlorizin		Improves the symptoms of diabetes and diabetic complications.		[104]
	dihydrochalcones from the roots of *Sophora flavescens*		Inhibitors of PTP1B	PTP1B inhibition assay	[105]
	hydroxydihydrochalcones from *Artemisia* (Astraceae)	spectrophotometric method	Have inhibitory activity against principal enzymes of carbohydrate metabolism, such as α-amylase (IC_50_ = 150.24–384.14 μg/mL) and α-glucosidase (IC_50_ = 214.42–754.12 μg/mL). May become a complement to synthetic antidiabetic drugs for controlling blood glucose level.	In vitro, α-glucosidase from *Saccharomyces cerevisiae*, α-amylase from *Aspergillus oryzae*	[106]
	aspalathin	130 mg/kg	Protects against diabetes-associated symptoms in mice.	In vivo, type-2 diabetic mouse model	[107]
		complications associated with hepatic insulin resistance and metabolic disease-relate	Plays a significant role in the maintenance of hormonal homeostasis. Influences the steroid hormone biosynthesis and the flux through the mineralocorticoid, glucocorticoid, and androgen pathways, thus possibly contributes to the alleviation of negative effects arising from elevated glucocorticoid levels.	In vitro, H295R	[108]
		10 µM	Reverses the palmitate-induced insulin resistance. Suppresses nuclear factor kappa beta (NF-κB), insulin receptor substrate one (serine 307) (IRS1 (Ser (307) and AMP-activated protein kinase (AKT) phosphorylation and increases serine/threonine kinase AKT activation. Increases the peroxisome proliferator-activated receptor alpha and gamma (PPARα and γ) and carnitine palmitoyltransferase one (CPT1) expression.	In vitro, 3T3-L1 adipocytes exposed to palmitate	[109]
		10 µM	Regulates hepatic cellular metabolism, increases energy expenditure, and modulates PI3K/AKT and AMPK signaling pathways.	In vitro, C3A (liver cells exposed to palmitate)	[110]
	phlorizin	5–40 mg/kg	Competitive inhibitor of sodium/glucose cotransporters in the intestine (SGLT1) and kidney (SGLT2) involved in intestinal glucose absorption and renal glucose reabsorption.	In vivo, Wistar rats	[111]
Kidney diseases	aspalathin and nothofagin		Induce renal damage. Inhibits nuclear factor (NF)-κB activation and reduces the induction of NO synthase and excessive production of nitric acid. Reduce the plasma levels of NO, TNF-α, and IL-6; increases lipid peroxidation; and markedly enhances the antioxidant defence system by restoring the levels of superoxide dismutase, glutathione peroxidase, and catalase.	In vivo, mice model sepsis	[112]
Antimicrobial activity	balsacones A, B, and C		MIC = 3.1–6.6 μM (*S. aureus*) resistance (*E. coli*)	In vitro, *Staphylococcus aureus* *Escherichia coli*	[113]
	Elastichalcone C			In vitro, methicillin-resistant *Staphylococcus aureus* SK1, *Saprolegnia parasitica Saprolegnia diclina*	[114]
	dihydroisorcordoin			In vitro, *Saprolegnia parasitica Saprolegnia diclina*	[115]
	phloretin, phlorizin,3’,5’-*di*-C-glucoside	microdilution method	phloretin: MIC = 7.81–125 μM (*S. aureus*) MIC = 62.5 μM (*L. monocytogenes*) MIC = 125 μM (*S. typhimurium*) MIC > 1000 μM (*P. aeruginosa*, *E. coli*)	In vitro, *Staphylococcus aureus* ATCC 6538 *Listeria monocytogenes* ATCC 13932 methicyllin-resistant *Staphylococcus aureus* (MRSA, clinical strains: 526, 588, 550, 541, 531, 530, 543, 581) *Salmonella typhimurium* ATCC 13311 *Pseudomonas aeruginosa* ATCC 27853 *Pseudomonas aeruginosa* ATCC 15442 *E. coli* ATCC 10536	[116]
	2′,4′-dihydroxy-4- methoxy-3′-prenyldihydrochalcone 2′,4′-dihydroxy-3,4-(2”,2”-dimethylchromeno)-3′-prenyldihydrochalcone		Inhibition zone: 7.25–10.75 mm MIC = 0.9–1.8 mg/mL	In vitro, *Bacillus subtilis* (ATCC 6633), *Staphylococcus aureus* (ATCC 29737), *Escherichia coli* (ATCC 10536), *Pseudomonas putida* (ATCC 49128)	[117]
	2′,4’-dihydroxychalcone	105 µg/mL	Inhibits spore germination of plant pathogenic fungi	In vitro, *Heiminthosporium oryzae, Aspergillus niger, Rhizopus nigricans*	[118]
	2′,6′-dihydroxy-3′-methyl-4′-methoxydihydrochalcone		MIC = 1.0–7.8 mg/L	In vitro, *B. cereus* (IFO3001) *Pseudomonas putida* (IFO3738) *S. aureus* (IFO12732) methicillin-resistant *S. aureus* (MRSA; RIM0310925) *Enterococcus faecalis* (IFO12970) *Alicyclobacillus acidoterrestris* (ATCC49025) *Propionibacterium acnes* (ATCC6919) *Trichophyton mentagrophytes* (IFO5466)	[119]
	dihydrochalcones identified in ethanol extracts from *Uvaria chamae* roots (uvaretin, uvangoletin, diuvaretin)		Inhibition diameter: 9–30.7 mm	*S. aureus* ATCC 259223 *S. aureus* (clinical isolate) *E. faecalis* (clinical isolate)	[120]
	aspalathin, nothofagin		Antibacterial effect	In vitro, *Escherichia coli* (CFT073) *Staphylococcus epidermis* (Se19) *Staphylococcus aureus* (ATCC 25923)	[121]
Antioxidant activity	neohesperidin dihydrochalcone		Downregulates cyclooxygenase-2 (COX-2) and inducible nitric oxide synthase (iNOS) expressions. Inhibites PQ-induced nuclear factor-kappa B (NF-κB) expression and mitochondrial-driven apoptotic signaling	In vivo, mice with paraquat (PQ)-induced acute liver injury	[122]
	phlorizin, trilobatin, 3-hydroxyphlorizin, sieboldin, phloretin 2′-xyloglucosid		3-Hydroxyphloretin was the best antioxidant among the seven compounds. Both glycosylation of the A ring and the ortho phenolic hydroxyl groups of the B ring were important for the cytotoxicity of dihydrochalcone molecules.	DPPH and ABTS assays	[123]
	phloretin, phlorizin, trilobatin, sieboldin, 3-hydroxyphloretin 3-hydroxyphlorizin		Presence of an *o*-dihydroxyl group in the B-ring increased activity, whereas glycosylation in the A-ring decreased antioxidant potential of dihydrochalcones. Glycosylation at the 2′-position enhanced the dissociation ability of the 4′-hydroxyl group and increased the antioxidant activity of dihydrochalcones containing o-dihydroxyl.	DPPH and ABTS assays	[124]
	phloretin, phloridzin, trilobatin, naringin dihydrochalcone, neohesperidin dihydrochalcone	1 mg/mL (2–10 µL) FRAP, DPPH 0.25 mg/mL (2–10 µL) ABTS 5 mg/mL (2–10 µL) superoxide radical scavenging	Antioxidant structure-activity relationship. In FRAP assay, antioxidant activity of pairs of dihydrochalcones had the following relationship: phloretin > phloridzin, phloretin > trilobatin, trilobatin > phloridzin, trilobatin > naringin dihydrochalcone, neohesperidin dihydrochalcone > naringin dihydrochalcone.	FRAP, DPPH, ABTS, and superoxide radical scavenging assays	[125]
	aspalathin, nothofagin		Inhibitor of Fe(II)-induced lipid peroxidation: IC_50_ = 50.2 µM (aspalathin) IC_50_ = 1388 µM (aspalathin) ABTS assay: IC_50_ = 3.33 µM (aspalathin) IC_50_ = 4.04 µM (nothofagin)	ABTS, metal chelating, and Fe(II)-induced microsomal lipid peroxidation assays	[126]
	trilobatin, hesperetin dihydrochalcone-7-*O*-glucoside, prunin and hesperetin-7-*O*-glucoside (obtained in the selective enzymatic hydrolysis of parental flavonoid glycosides using an immobilised α-L-rhamnosidase)	0.4–1.0 mM (DPPH) 0.025–0.200 mM (FRAP) 20–80 µM (ORAC)	Some of the flavonoid monoglucosides showed significant improvement in the antioxidant activity.	DPPH, FRAP, and ORAC assays	[127]
	flavanones and dihydrochalcones		Dihydrochalcones exhibited higher antioxidant activities than the corresponding flavanones. The hydroxyl group at the 2‘-position in dihydrochalcone A ring is an essential pharmacophore for its radical scavenging potential.	DPPH assay and lipid peroxidation in the erythrocyte membrane assays	[128]
	phloretin	1–5 mM	Supresses lipid oxidation in PUFA % of inhibition of the peroxyl radical-induced oxidation in oil-in-water emulsion containing methyl linolenate = 72.5 ± 5.5	In vitro, oil–in–water emulsion system, TBARS and fish oil system	[129]
Antiinflammatory activity	phloretin	125 µM	IC_50_ = 20 µM (MMP-2) does not inhibit LE and MMP-9	In vitro, matrix-proteases, leukocyte elastase (LE), and gelatinase A (MMP-2; human neuroblastoma cells) gelatinase B (MMP-9; HT-1080 human fibrosarcoma cells)	[130]
	aspalathin nothofagin		Inhibits HG-mediated vascular hyperpermeability, adhesion of monocytes toward HUVECs, and expression of CAMs. Suppresses the formation of ROS and the activation of NFκB.	In vivo, male C57BL/6 mice In vitro, primary human umbilical vein endothelial cells (HUVECs)	[131]
	α,β-dihydroxantohumol	1–15 µM	IC_50_ (COX-1) = 124.50 ± 7.61 IC_50_ (COX-2) = 103.8 ± 6.11	In vitro, binding to human serum albumin (HSA), COX-1, and COX-2 activity	[132]
	phloretin	Osteoarthritis (OA)	Inhibits the IL-1β-induced production of NO, PGE2, TNF-α, and IL-6; the expression of COX-2, iNOS, MMP-3, MMP-13, and ADAMTS-5; and the degradation of aggrecan and collagen-II in human chondrocytes. Suppresses the IL-1β-stimulated phosphorylation of PI3K/Akt and activation of NF-κB. Decreass the expression of MMP-13 and increases the expression of collagen-II in mice.	In vivo, mice In vitro, human OA chondrocytes	[133]
	aspalathin nothofagin	MTT assay 9.1–27.1 µg/mouse (aspalathin) 8.7–26.2 µg/mouse (nothofagin)	Inhibit LPS-induced barrier disruption, expression of cell adhesion molecules (CAMs), and adhesion/transendothelial migration of neutrophils to human endothelial cells. Suppress LPS-induced hyperpermeability and leukocyte migration. Suppress the production of tumor necrosis factor-α (TNF-α) or interleukin (IL)-6 and the activation of nuclear factor-κB (NF-κB) or extracellular regulated kinases (ERK) 1/2 by LPS	In vitro, HUVECs (primary human umbilical vein endothelial cells) In vivo, male C57BL/c mice	[134]
	apple polyphenol		Significantly inhibits proinflammatory gene expression and represses NF-jB-, IP-10-, IL-8-promoter-, and STAT1-dependent signal transduction in a dose-dependent manner.	In vitro, T84 cells (colon epithelial cells), the human colon adenocarcinoma 1264 cell line DLD-1 (DSMZ ACC278), the human leukemia T-cell line Jurkat (DSMZ ACC282), and the human monocyticleukemia cell lineMonoMac6 (DSMZ ACC124)	[135]
Hepatoprotective	phloretin	50 mg/kg	Reduces mortality rate in mice, resulting in protection against lethal effect of acetaminophen. Downregulates SGOT serum transaminases.	In vivo, mice with hepatotoxicity induced by acetaminophen	[136]
	2′,4′-dihydroxydihydrochalcone-4-*O*-β-D-glucopyranoside	MTT assay 10 µM	Cell survival rate (% of normal) 74.63 ± 11.11	In vitro, D-galactosamine-induced toxicity inhuman hepatoma HepG2 cells	[137]
Antileishmanial activity			IC_50_ = 2.98 µg/mL IC_50_ = 3.65 µg/mL	In vitro, promastigotes of *Leishmania braziliensis*	[138]
Anti-HIV, anti-herpes simplex virus type 2	trilobatin		Exhibits broad anti-HIV activity and displays synergistic anti-HIV activities combined with antiretroviral agents (e.g., maraviroc, zidovudine, and raltegravir). Effective in inhibiting HSV-2 (at the concetration of 458 µM)	In vitro, MT-2 and Vero cells	[139]
Cardioprotective	aspalathin	1 µM 13 and 130 mg/kg	Protects cardiomyocytes against hyperglycemia-induced shifts in substrate preference and subsequent apoptosis; prevents myocardium apoptosis; modulates key regulators associated with lipid metabolism (Adipoq, Apob, CD36, Cpt1, Pparγ, Srebf1/2, Scd1, and Vldlr), insulin resistance (Igf1, Akt1, Pde3, and Map2k1), inflammation (Il3, Il6, Jak2, Lepr, Socs3, and Tnf13), and apoptosis (Bcl2 and Chuk)	In vitro, H9c2 cardiomiocytes In vivo, C57BLKS/J homozygous Lepr^db/db^ mice	[140]
Anticholinesterase and antibutyrylcholinesterases activity	phloretin phlorizin	0.2 mM	Exhibit anti-AChE and anti-BChEs activity. Aglycons were more effective than corresponding glucosides.	In vitro, acetylcholinesterase (AChE, C3389), butyrylcholinesterase (BChE, C7512)	[141]
